# Supplementary material for: Exploration of a miRNA-mRNA network shared between acute pancreatitis and Epstein-Barr virus infection by integrated bioinformatics analysis
Source: PLoS One. 2024 Nov 15;19(11):e0311130. doi: 10.1371/journal.pone.0311130 (PMC11567522; doi:10.1371/journal.pone.0311130)
Supplement: S1 Table — (DOCX) [file pone.0311130.s001.docx]

**S1 Table. Detailed information about the platform and sample information of the included datasets (GSE194331, GSE45918, GSE42455 and GSE109220).**

| Dataset | Title | Organism | Source | Platform | PMID |
| --- | --- | --- | --- | --- | --- |
| GSE194331 | Gene Expression Profiling of Acute Pancreatitis | Homo sapiens | Peripheral blood | GPL16791 | 35426393 |
| GSE45918 | Peripheral blood gene expression in human experiencing primary EBV infection (Ref8) | Homo sapiens | Peripheral blood | GPL6883 | 24465555 |
| GSE42455 | microRNA expression in Mestenteric lymph samples from rat models of acute pancreatitis | Rattus norvegicus | Mestenteric lymph | GPL8786 | 24509209 |
| GSE109220 | miRNA expression data from human PBMC derived from acute infectious mononucleosis patients | Homo sapiens | Peripheral blood | GPL19117 | 29379474 |
