# Supplementary material for: Exploration of a miRNA-mRNA network shared between acute pancreatitis and Epstein-Barr virus infection by integrated bioinformatics analysis
Source: PLoS One. 2024 Nov 15;19(11):e0311130. doi: 10.1371/journal.pone.0311130 (PMC11567522; doi:10.1371/journal.pone.0311130)
Supplement: S2 Table — (DOCX) [file pone.0311130.s002.docx]

**S2 Table. 111 overlapping DEGs between datasets GSE194331 and GSE45918.**

|  | Gene | Type |  | Gene | Type |
| --- | --- | --- | --- | --- | --- |
| 1 | UBE2C | Up | 57 | STRBP | Down |
| 2 | TBKBP1 | Up | 58 | SPRY1 | Down |
| 3 | STK16 | Up | 59 | SPON1 | Down |
| 4 | SPAG6 | Up | 60 | SPOCK2 | Down |
| 5 | SIRPB2 | Up | 61 | SPIB | Down |
| 6 | PSMA4 | Up | 62 | SLC16A10 | Down |
| 7 | POLR2J | Up | 63 | SH3YL1 | Down |
| 8 | PLSCR1 | Up | 64 | SDK2 | Down |
| 9 | PKMYT1 | Up | 65 | SALL2 | Down |
| 10 | PELO | Up | 66 | RGMB | Down |
| 11 | PARP9 | Up | 67 | RAB11FIP5 | Down |
| 12 | NMI | Up | 68 | PLXNA1 | Down |
| 13 | NAT1 | Up | 69 | PLEKHB1 | Down |
| 14 | MXD3 | Up | 70 | PLEKHA1 | Down |
| 15 | MRPL51 | Up | 71 | PLAG1 | Down |
| 16 | LMNB1 | Up | 72 | PDE9A | Down |
| 17 | LDHA | Up | 73 | OSBPL10 | Down |
| 18 | KIF4A | Up | 74 | OLFM1 | Down |
| 19 | JAK2 | Up | 75 | NRCAM | Down |
| 20 | IFI27 | Up | 76 | NR3C2 | Down |
| 21 | IFI16 | Up | 77 | NLGN2 | Down |
| 22 | HMMR | Up | 78 | NCAM1 | Down |
| 23 | HMGB3 | Up | 79 | LRRN3 | Down |
| 24 | HMGB2 | Up | 80 | LDLRAP1 | Down |
| 25 | HAT1 | Up | 81 | KLHL3 | Down |
| 26 | GPSM2 | Up | 82 | KIR2DL1 | Down |
| 27 | GGH | Up | 83 | IL7R | Down |
| 28 | GBP2 | Up | 84 | IGFBP3 | Down |
| 29 | FBXO6 | Up | 85 | HOOK1 | Down |
| 30 | FAS | Up | 86 | GSTM3 | Down |
| 31 | EXOSC4 | Up | 87 | FMNL3 | Down |
| 32 | E2F2 | Up | 88 | FCRL3 | Down |
| 33 | CTNNAL1 | Up | 89 | FCGBP | Down |
| 34 | CEP55 | Up | 90 | EPHX2 | Down |
| 35 | CDC25C | Up | 91 | DSC1 | Down |
| 36 | C2 | Up | 92 | DHRS3 | Down |
| 37 | C1QC | Up | 93 | DBNDD1 | Down |
| 38 | C1QB | Up | 94 | CRIP2 | Down |
| 39 | C1QA | Up | 95 | CR2 | Down |
| 40 | BATF | Up | 96 | COBLL1 | Down |
| 41 | B4GALT5 | Up | 97 | CNN3 | Down |
| 42 | ANKRD22 | Up | 98 | CD79A | Down |
| 43 | ZNF573 | Down | 99 | CD40LG | Down |
| 44 | ZNF365 | Down | 100 | CCR7 | Down |
| 45 | ZNF135 | Down | 101 | CAMK2N1 | Down |
| 46 | ZNF10 | Down | 102 | CACNA2D2 | Down |
| 47 | WDR27 | Down | 103 | BIRC3 | Down |
| 48 | VSIG1 | Down | 104 | BANK1 | Down |
| 49 | VPREB3 | Down | 105 | BACH2 | Down |
| 50 | TSPAN13 | Down | 106 | AXIN2 | Down |
| 51 | TRPC1 | Down | 107 | AKT3 | Down |
| 52 | TNFRSF21 | Down | 108 | AK5 | Down |
| 53 | TMEM30B | Down | 109 | AHNAK | Down |
| 54 | TJP3 | Down | 110 | AFF3 | Down |
| 55 | TEAD2 | Down | 111 | ABCB1 | Down |
| 56 | TBC1D4 | Down |  |  |  |
