# Supplementary material for: Exploration of a miRNA-mRNA network shared between acute pancreatitis and Epstein-Barr virus infection by integrated bioinformatics analysis
Source: PLoS One. 2024 Nov 15;19(11):e0311130. doi: 10.1371/journal.pone.0311130 (PMC11567522; doi:10.1371/journal.pone.0311130)
Supplement: S3 Table — (DOCX) [file pone.0311130.s003.docx]

**S3 Table. Enrichment analysis results based on 42 overlapping up-regulated DEGs.**

| ID | ONTOLOGY | Description | p.adjust | geneID | Count |
| --- | --- | --- | --- | --- | --- |
| GO:0098883 | BP | Synapse pruning | 9.39E-04 | C1QC/C1QB/C1QA | 3 |
| GO:0009615 | BP | Response to virus | 9.39E-04 | TBKBP1/PLSCR1/PARP9/NMI/JAK2/IFI27/IFI16/EXOSC4 | 8 |
| GO:0045089 | BP | Positive regulation of innate immune response | 3.77E-03 | PLSCR1/PARP9/NMI/IFI16/HMGB2 | 5 |
| GO:0150146 | BP | Cell junction disassembly | 3.77E-03 | C1QC/C1QB/C1QA | 3 |
| GO:0060333 | BP | Interferon-gamma-mediated signaling pathway | 4.40E-03 | PARP9/NMI/JAK2 | 3 |
| GO:0051607 | BP | Defense response to virus | 5.08E-03 | TBKBP1/PLSCR1/PARP9/IFI27/IFI16/EXOSC4 | 6 |
| GO:0140546 | BP | Defense response to symbiont | 5.08E-03 | TBKBP1/PLSCR1/PARP9/IFI27/IFI16/EXOSC4 | 6 |
| GO:0031349 | BP | Positive regulation of defense response | 5.08E-03 | PLSCR1/PARP9/NMI/JAK2/IFI16/HMGB2 | 6 |
| GO:0002833 | BP | Positive regulation of response to biotic stimulus | 5.08E-03 | PLSCR1/PARP9/NMI/IFI16/HMGB2 | 5 |
| GO:0006958 | BP | Complement activation, classical pathway | 7.29E-03 | C2/C1QC/C1QB/C1QA | 4 |
| GO:0016064 | BP | Immunoglobulin mediated immune response | 7.68E-03 | C2/C1QC/C1QB/C1QA/BATF | 5 |
| GO:0019724 | BP | B cell mediated immunity | 7.68E-03 | C2/C1QC/C1QB/C1QA/BATF | 5 |
| GO:0071346 | BP | Cellular response to interferon-gamma | 8.16E-03 | PARP9/NMI/JAK2/GBP2 | 4 |
| GO:0002455 | BP | Humoral immune response mediated by circulating immunoglobulin | 8.16E-03 | C2/C1QC/C1QB/C1QA | 4 |
| GO:0002460 | BP | Adaptive immune response based on somatic recombination of immune receptors built from immunoglobulin superfamily domains | 8.46E-03 | JAK2/C2/C1QC/C1QB/C1QA/BATF | 6 |
| GO:0002274 | BP | Myeloid leukocyte activation | 8.51E-03 | PLSCR1/NMI/JAK2/C1QA/BATF | 5 |
| GO:0045088 | BP | Regulation of innate immune response | 8.51E-03 | PLSCR1/PARP9/NMI/IFI16/HMGB2 | 5 |
| GO:0006956 | BP | Complement activation | 9.10E-03 | C2/C1QC/C1QB/C1QA | 4 |
| GO:0002253 | BP | Activation of immune response | 9.10E-03 | PLSCR1/IFI16/C2/C1QC/C1QB/C1QA | 6 |
| GO:2001269 | BP | Positive regulation of cysteine-type endopeptidase activity involved in apoptotic signaling pathway | 9.42E-03 | JAK2/FAS | 2 |
| GO:0030099 | BP | Myeloid cell differentiation | 9.42E-03 | JAK2/IFI16/HMGB3/HMGB2/C1QC/BATF | 6 |
| GO:0034341 | BP | Response to interferon-gamma | 9.71E-03 | PARP9/NMI/JAK2/GBP2 | 4 |
| GO:0070936 | BP | Protein K48-linked ubiquitination | 1.66E-02 | UBE2C/NMI/IFI27 | 3 |
| GO:0032103 | BP | Positive regulation of response to external stimulus | 1.66E-02 | PLSCR1/PARP9/NMI/JAK2/IFI16/HMGB2 | 6 |
| GO:0071025 | BP | RNA surveillance | 2.05E-02 | PELO/EXOSC4 | 2 |
| GO:0019221 | BP | Cytokine-mediated signaling pathway | 2.15E-02 | TBKBP1/PARP9/NMI/JAK2/IFI27/FAS | 6 |
| GO:2001267 | BP | Regulation of cysteine-type endopeptidase activity involved in apoptotic signaling pathway | 2.15E-02 | JAK2/FAS | 2 |
| GO:0016075 | BP | RRNA catabolic process | 3.19E-02 | PELO/EXOSC4 | 2 |
| GO:0002831 | BP | Regulation of response to biotic stimulus | 3.23E-02 | PLSCR1/PARP9/NMI/IFI16/HMGB2 | 5 |
| GO:0002449 | BP | Lymphocyte mediated immunity | 3.36E-02 | C2/C1QC/C1QB/C1QA/BATF | 5 |
| GO:0097191 | BP | Extrinsic apoptotic signaling pathway | 4.04E-02 | JAK2/IFI27/HMGB2/FAS | 4 |
| GO:0042832 | BP | Defense response to protozoan | 4.62E-02 | GBP2/BATF | 2 |
| GO:0042116 | BP | Macrophage activation | 4.68E-02 | NMI/JAK2/C1QA | 3 |
| GO:0001562 | BP | Response to protozoan | 4.68E-02 | GBP2/BATF | 2 |
| hsa04936 | KEGG | Alcoholic liver disease | 4.47E-03 | FAS/C2/C1QC/C1QB/C1QA | 5 |
| hsa05133 | KEGG | Pertussis | 4.47E-03 | C2/C1QC/C1QB/C1QA | 4 |
| hsa04610 | KEGG | Complement and coagulation cascades | 4.77E-03 | C2/C1QC/C1QB/C1QA | 4 |
| hsa05150 | KEGG | Staphylococcus aureus infection | 5.26E-03 | C2/C1QC/C1QB/C1QA | 4 |
| hsa05142 | KEGG | Chagas disease | 5.26E-03 | FAS/C1QC/C1QB/C1QA | 4 |
| hsa05322 | KEGG | Systemic lupus erythematosus | 1.37E-02 | C2/C1QC/C1QB/C1QA | 4 |
| hsa04148 | KEGG | Efferocytosis | 1.81E-02 | JAK2/C1QC/C1QB/C1QA | 4 |
