# Supplementary material for: Exploration of a miRNA-mRNA network shared between acute pancreatitis and Epstein-Barr virus infection by integrated bioinformatics analysis
Source: PLoS One. 2024 Nov 15;19(11):e0311130. doi: 10.1371/journal.pone.0311130 (PMC11567522; doi:10.1371/journal.pone.0311130)
Supplement: S4 Table — (DOCX) [file pone.0311130.s004.docx]

**S4 Table. Enrichment analysis results based on 69 overlapping down-regulated DEGs.**

| ID | ONTOLOGY | Description | p.adjust | geneID | Count |
| --- | --- | --- | --- | --- | --- |
| GO:0042100 | BP | B cell proliferation | 2.39E-03 | TNFRSF21/IL7R/FCRL3/CR2/CD79A/CD40LG | 6 |
| GO:0002768 | BP | Immune response-regulating cell surface receptor signaling pathway | 1.71E-02 | TNFRSF21/PLEKHA1/KIR2DL1/FCRL3/CR2/CD79A/CCR7/BANK1 | 8 |
| GO:0002429 | BP | Immune response-activating cell surface receptor signaling pathway | 3.78E-02 | TNFRSF21/PLEKHA1/FCRL3/CR2/CD79A/CCR7/BANK1 | 7 |
| GO:0002757 | BP | Immune response-activating signal transduction | 3.78E-02 | TNFRSF21/PLEKHA1/FCRL3/CR2/CD79A/CCR7/BANK1 | 7 |
| hsa05340 | KEGG | Primary immunodeficiency | 4.31E-02 | IL7R/CD79A/CD40LG | 3 |
| hsa04662 | KEGG | B cell receptor signaling pathway | 4.31E-02 | CR2/CD79A/BANK1/AKT3 | 4 |
| DOID:2377 | Disease | Multiple sclerosis | 1.69E-02 | IL7R/IGFBP3/GSTM3/CR2/CD40LG/CCR7/BIRC3 | 7 |
| DOID:3213 | Disease | Demyelinating disease | 1.69E-02 | IL7R/IGFBP3/GSTM3/CR2/CD40LG/CCR7/BIRC3 | 7 |
| DOID:1168 | Disease | Familial hyperlipidemia | 1.70E-02 | OSBPL10/LDLRAP1/EPHX2/CD40LG/ABCB1 | 5 |
| DOID:3969 | Disease | Thyroid gland papillary carcinoma | 1.70E-02 | NRCAM/NCAM1/DHRS3/CCR7/CAMK2N1 | 5 |
| DOID:526 | Disease | Human immunodeficiency virus infectious disease | 1.70E-02 | KIR2DL1/IL7R/CR2/CD40LG/BACH2/ABCB1 | 6 |
| DOID:0080524 | Disease | Thyroid gland adenocarcinoma | 1.70E-02 | NRCAM/NCAM1/DHRS3/CCR7/CAMK2N1 | 5 |
| DOID:0080525 | Disease | Differentiated thyroid gland carcinoma | 1.70E-02 | NRCAM/NCAM1/DHRS3/CCR7/CAMK2N1 | 5 |
| DOID:0060100 | Disease | Musculoskeletal system cancer | 2.92E-02 | SPRY1/SALL2/PLXNA1/IL7R/IGFBP3/GSTM3/COBLL1/ABCB1 | 8 |
| DOID:3146 | Disease | Lipid metabolism disorder | 3.64E-02 | OSBPL10/LDLRAP1/EPHX2/CD40LG/ABCB1 | 5 |
| DOID:201 | Disease | Connective tissue cancer | 3.64E-02 | SALL2/PLXNA1/IL7R/IGFBP3/GSTM3/COBLL1/ABCB1 | 7 |
| DOID:5158 | Disease | Pleural cancer | 3.64E-02 | PLXNA1/IL7R/COBLL1 | 3 |
| DOID:7474 | Disease | Malignant pleural mesothelioma | 3.64E-02 | PLXNA1/IL7R/COBLL1 | 3 |
| DOID:0060060 | Disease | Non-Hodgkin lymphoma | 4.01E-02 | NCAM1/KIR2DL1/CD40LG/ABCB1 | 4 |
| DOID:4479 | Disease | Pseudohypoaldosteronism | 4.01E-02 | NR3C2/KLHL3 | 2 |
| DOID:2583 | Disease | Agammaglobulinemia | 4.33E-02 | CR2/CD79A/CD40LG | 3 |
