# Supplementary material for: Exploration of a miRNA-mRNA network shared between acute pancreatitis and Epstein-Barr virus infection by integrated bioinformatics analysis
Source: PLoS One. 2024 Nov 15;19(11):e0311130. doi: 10.1371/journal.pone.0311130 (PMC11567522; doi:10.1371/journal.pone.0311130)
Supplement: Attachment — Submitted filename: Rebuttal letter.docx [file pone.0311130.s009.docx]

**PONE-D-24-30500**

**Exploration of a miRNA-mRNA network shared between acute pancreatitis and Epstein-Barr virus infection by integrated bioinformatics analysis**

Dear editor and reviewers:

Thank you for your decision and constructive comments on our manuscript. We have carefully considered the comments and tried our best to address every one of them. According to your comments, revision notes, point-to-point, are given as follows:

**Journal Requirements**

1. When submitting your revision, we need you to address these additional requirements. Please ensure that your manuscript meets PLOS ONE's style requirements, including those for file naming. The PLOS ONE style templates can be found at

https://journals.plos.org/plosone/s/file?id=wjVg/PLOSOne_formatting_sample_main_body.pdf and

https://journals.plos.org/plosone/s/file?id=ba62/PLOSOne_formatting_sample_title_authors_affiliations.pdf

**Response:**

Thank you very much for your professional advice. We have improved the manuscript style based on the PLOS ONE style templates.

2. We note that the grant information you provided in the ‘Funding Information’ and ‘Financial Disclosure’ sections do not match. When you resubmit, please ensure that you provide the correct grant numbers for the awards you received for your study in the ‘Funding Information’ section.

**Response:**

Thank you very much for your professional advice. We try to add detailed grant information in the ‘Financial Disclosure’ section to be consistent with the description in the ‘Funding Information’ section. But the existing submission platform lacks the ‘Financial Disclosure’ section in the Questionnaire during the Additional Information phase, thereby precluding any modifications on our part. As a result, in the ‘Financial Disclosure’ section of final built PDF it says The author(s) received no specific funding for this work. We would greatly appreciate it if you could guide us to resolve the above issues. Thank you again for your consideration and kind assistance.

3. Please note that your Data Availability Statement is currently missing [the repository name and/or a direct link to access each database]. If your manuscript is accepted for publication, you will be asked to provide these details on a very short timeline. We therefore suggest that you provide this information now, though we will not hold up the peer review process if you are unable.

**Response:**

Thank you very much for your professional advice. The Data Availability Statement has been supplemented as follows: All datasets are available from the GEO database (GSE194331, GSE42455, GSE45918 and GSE109220). All relevant data are within the paper and its supporting information files.

4. Your ethics statement should only appear in the Methods section of your manuscript. If your ethics statement is written in any section besides the Methods, please delete it from any other section.

**Response:**

Thank you very much for your professional advice. The ethics statement has been supplemented in ‘Data collection’ section as follows: This study is based on the public datasets in the GEO database, which does not contain the specific information of the subjects and their personal privacy. Therefore, we believe that this study does not require ethics approval.

**Reviewer #1**

**Reviewer #1:** The manuscript titled "Exploration of a miRNA-mRNA network shared between acute pancreatitis and Epstein-Barr virus infection by integrated bioinformatics analysis" presents an interesting study that delves into the shared molecular mechanisms between acute pancreatitis (AP) and Epstein-Barr virus (EBV) infection. The use of integrated bioinformatics analysis to identify miRNA-mRNA networks is commendable, and the study's findings could contribute to a deeper understanding of these pathological conditions. However, several areas need improvement to enhance the manuscript's clarity, rigor, and overall impact.

Firstly, the manuscript would benefit from a more detailed explanation of the methods used in the differential expression analysis and network construction. While the authors have provided a general overview, the specific criteria for selecting differentially expressed genes (DEGs) and miRNAs (DEMs) and the rationale behind these choices need to be more clearly articulated. Additionally, the process of intersecting DEGs and DEMs to construct the miRNA-mRNA network should be described with greater precision, including any assumptions made or thresholds applied.

**Response:**

We are so grateful for your kind question. Differential expression analysis is currently one of the mainstream analytical methods for finding disease-related molecules. The package DESeq2^[1]^ provides methods to test for differential expression by use of negative binomial generalized linear models on un-normalized RNA-seq counts data. Limma^[2]^ is an R package for the analysis of gene expression data, especially the use of linear models for assessment of differential expression on normalized microarray data. DEGs or DEMs mean that there is a statistically significant difference in the expressions between two groups of samples. The choice of the specific cutoff value will generally be based on the experimental design and the objectives of the study. The cutoff value of fold change is usually set to 1.3^[3]^, 1.5^[4]^ or 2^[5]^, with a corrected p-value < 0.05^[6]^, sometimes a p-value < 0.05^[7]^. In order to adequately identify more DEGs and DEMs in the present study, we set the cutoff value of fold change to 1.5, meaning the differential gene or miRNA for which the expression level of the disease group samples was up- or down-regulated by a factor of 1.5 compared with that of the control samples, and it is also necessary to satisfy the corrected p-value < 0.05 in the datasets GSE194331, GSE45918 and GSE109220. Moreover, a slight adjustment was made to the threshold criteria of significance level, setting it at a p-value < 0.05 within the dataset GSE42455. This was because only 11 DEMs were identified using the corrected p-value < 0.05 as the threshold.

The get_multimir() function of the R package multiMiR^[8]^ was employed to retrieve the DEMs target genes^[9]^. The multiMiR contains a wide collection of validated and predicted miRNA–target interactions and their associations with drugs and diseases. It is composed of 14 external databases, which include 3 validated, 8 predicted, and 3 drug- or disease-related miRNA-target databases. In order to more accurately predict target genes of a given miRNA, we only considered validated miRNA-target interactions from three databases (miRecords, miRTarBase and TarBase). According to the detailed information provided by the multiMiR website (http://multimir.org/), there are 3045, 595913 and 644186 miRNA-target interactions records from miRecords, miRTarBase and TarBase, respectively. Given that the miRecords database contains approximately 3000 records, significantly fewer than miRTarBase and TarBase, and has not been updated since 2013, we opted to focus on miRNA-target gene interactions present in a minimum of two databases instead of requiring overlap across all three databases to ascertain the ultimate target genes.

Using the 8 overlapping DEMs as input, a total of 25, 1057, and 1055 validated miRNA-target interactions were found in miRecords, miRTarBase, and TarBase databases by multiMiR package, respectively (Supporting information, S6 Table). Then 960 target genes identified in at least two databases were further selected. A total of 10 overlapping genes were identified by taking intersections in 960 target genes and 111 DEGs. Finally, a miRNA-mRNA network of 11 miRNA–mRNA interaction pairs was constructed, which included 5 DEMs: miR-24-3p, miR-23b-3p, miR-130a-3p, miR-324-5p, miR-497-5p, and 10 DEGs: E2F2, HMGB2, LDHA, UBE2C, LMNB1, CEP55, AHNAK, AKT3, PLAG1, PLEKHA1.

**Reviewer #1:** The enrichment analysis results, while comprehensive, could be more effectively integrated into the narrative. Currently, the findings from the Gene Ontology (GO), Kyoto Encyclopedia of Genes and Genomes (KEGG), and disease ontology analyses are presented in a somewhat fragmented manner. It would be beneficial to discuss these results in the context of the broader biological implications and how they relate to the known pathophysiology of AP and EBV infection. Furthermore, the study would benefit from a more critical discussion of the limitations of enrichment analysis, particularly the potential for over-interpretation of results due to the inherent biases in the databases used.

**Response:**

Thank you very much for your comments and professional advice. Enrichment analysis helps researchers gain mechanistic insight into gene or miRNA lists generated from genome-scale experiments. In this study, at the threshold for adjusted p-value < 0.05, we performed enrichment analysis of genes and miRNAs using the R package clusterProfiler^[10]^ and the miRNA Enrichment Analysis and Annotation Tool^[11]^ (miEAA, version 2.1), respectively. We have further summarized the results of the enrichment analysis in the Results section as follows: Utilizing the 42 up-regulated DEGs as inputs, a collective of 34 biological process (BP) and 7 KEGG terms were significantly enriched, mainly comprising innate and adaptive immune response, interferon-gamma, complement and apoptotic signaling pathways. Using the 69 down-regulated DEGs as input, a total of 4 BP, 2 KEGG and 15 disease ontology terms were significantly enriched, which mainly included B cell proliferation, immune response, autoimmune diseases and tumors. Using the miEAA tool, a total of 234 subcategories were significantly enriched based on 8 DEMs. GO and KEGG subcategories mainly include cell cycle, nutrient metabolism and organ development. The diseases subcategories mainly encompass a wide range of solid and hematological tumors, neurological disorders and autoimmune diseases.

The discussion and limitations of the enrichment analysis results has also been supplemented in the third paragraph of the Discussion section as follows: Functional enrichment analysis showed that shared DEGs and DEMs between AP and EBV infection were involved in various biological processes and signaling pathways, such as innate and adaptive immune responses, autophagy and apoptosis signaling pathways. Innate immunity is the first line of antiviral defense and the activation of interferon signaling pathway is one of the major antiviral defense mechanisms of host cells after EBV infection^[12]^. Consistent with our enrichment analysis results, a recent study also revealed that AP mice were enriched in biological processes related to immune system pathways and inflammatory responses^[13]^. However, innate immunity is also a double-edged sword, as the induction of pro-inflammatory responses and the activation of programmed cell death^[14]^. The pathophysiology of EBV-associated pancreatitis is unclear, but the pancreas is likely one of the target organs affected by the immune response. The unobstructed autophagy process is the basis for cells to maintain their metabolic functions, and the abnormal autophagy can cause autophagic vesicles to accumulation and lysosome inactivation, which is closely related to the occurrence of pancreatitis^[15]^. Studies also find that EBV have evolved multiple strategies to interfere with autophagy to avoid destruction and promote their own replication and spread^[16],^^[17]^. Increasing evidences indicate that EBV miRNAs and latent genes involved in manipulation of the cell apoptosis^[18],^^[19]^. Mild acute pancreatitis was found to be associated with extensive apoptotic acinar cell death, whereas severe AP was found to involve extensive acinar cell necrosis but very little acinar cell apoptosis^[20]^. Consistent with this, the results of our enrichment analysis found that apoptosis, but not necrosis, was significantly enriched. And the patients with EBV-associated AP were characterized by a favorable prognosis, without severe complications. However, since the dataset used for DEG and DEM identification are not from pancreatic tissue, it is still unclear which exact molecules and pathways are affected in the pancreas during EBV infection, and whether EBV can directly cause pancreatic cell damage and its potential molecular mechanisms still require further co-culture and in vivo studies.

**Reviewer #1:** The construction of the ceRNA and TF-miRNA-mRNA networks is an important aspect of the study. However, the manuscript would be strengthened by a more detailed discussion of the biological relevance of the identified networks. Specifically, the authors should provide more insight into the potential functional roles of the miRNAs and genes within these networks, as well as any known associations with AP and EBV infection. Additionally, the criteria for selecting the top 10 lncRNAs and circRNAs in the ceRNA network should be clarified, as this selection appears somewhat arbitrary.

**Response:**

We thank the reviewer for raising this question. ENCORI/starBase^[21]^ (Encyclopedia of RNA Interactomes) is an openly licensed and state-of-the-art platform to facilitate the integrative, interactive and versatile display of, as well as the comprehensive annotation and discovery of, RNA-RNA and protein-RNA interactions by deeply mining thousands of high-throughput sequencing data of RNA-RNA interactome, CLIP-seq and degradome sequencing. By inputting specific miRNA, miRNA-mRNA, miRNA-lncRNA, and miRNA-circRNA interactions predictions can be performed step by step. This highlights its prominence in ceRNA network construction studies^[22],^^[23],^^[24]^. We have added detailed information on miRNA-target interactions predictions in the supplementary materials in order to present the predictions results comprehensively and objectively (S7 Table). After aggregation, however, there are thousands of interactions predictions. In order to simplify the interaction links for visualization, the interactions results were sorted by the ClipExpNum^[25]^, which represented the number of supported experiments, with low number indicating weak interaction with the miRNA. Therefore, the corresponding top 10 lncRNAs and circRNAs candidates were selected for ceRNA network construction and visualization.

In the meantime, the discussion on the biological relevance of the identified networks has been supplemented in the fourth paragraph of the Discussion section as follows: In order to showcase the upstream and downstream regulatory relationships of hub molecules, the ceRNA and TF-miRNA-mRNA networks were constructed. We found that lncRNA NEAT1 exhibited close relationships with five identified hub miRNAs in the ceRNA network. There are no reported associations between NEAT1 and EBV infection, but studies have shown that NEAT1 has opposite effects in limiting the replication of dengue^[26]^ and Hantaan viruses^[27]^. Downregulation of NEAT1 relieves caerulein-induced cell apoptosis and inflammatory injury in pancreatic acinar cells through sponging miR-365a-3p^[28]^. The NEAT1/miR-130/IRF1 axis in gastric cancer^[29]^, NEAT1/miR-24-3p/LRG1 axis in thoracic aortic aneurysm^[30]^, NEAT1/miR-324-5p/RAN axis in retinoblastoma^[31]^ and NEAT1/miR-497-5p/PIK3R1 axis in renal tubular oxidative injury^[32]^ have been widely studied. E2F1 acts as a master regulator in the TF-miRNA-mRNA network, which is a member of the E2F family of transcription factors and plays a crucial role in the control of cell cycle and action of tumor suppressor proteins. Studies have found that EBV-encoded proteins induce activation of E2F1 and S-phase entry, which may facilitate efficient lytic EBV replication in certain cell types^[33],^^[34]^. A pan-cancer study indicated that KAT2A was found to cooperate with E2F1 and be recruited by E2F1 to the UBE2C promoter for elevating the expression of UBE2C by increasing the acetylation level of H3K9 to promote cell proliferation and the migration of cancer cells^[35]^. And increased E2F1 expression via the E2F1/FTH1P3/miR-377-3p/LDHA axis promoted cell viability and glycolysis in laryngeal squamous cell carcinoma^[36]^. In summary, although some studies have demonstrated the regulatory relationships in the ceRNA and TF-miRNA-mRNA networks, they still need to be further explored in the context of EBV infection and AP.

In addition, the potential functional roles of miRNAs and genes, as well as any known associations with AP and EBV infection were supplemented in the seventh and eighth paragraphs of the Discussion, respectively. Specifically, Su et al. reported that an miR-24-3p/MARCH3/NLRP3 axis mediated peritoneal macrophage M1 polarization and pyroptosis in AP^[37]^. With the exception of miR-24-3p, there are no reports that the other four miRNAs are associated with AP or EBV infection; however, the studies^[38],^^[39],^^[40],^^[41],^^[42],^^[43]^ highlight the important role of these miRNAs in regulating inflammatory responses and provide a theoretical basis for further research in the context of AP or EBV infection. A sum of 10 shared genes were encapsulated in the miRNA-mRNA network. Among them, three genes (HMGB2^[44]^, LDHA^[45],^^[46],^^[47]^ and AKT3^[48]^) were reported to be closely related to pancreatitis, and no gene was reported to be associated with EBV infection. However, these genes are directly or indirectly involved in the molecular events of AP and EBV infection, such as cell cycle regulation, the Ca^2+^ and Wnt signaling pathways and lymphocyte activation, and provide new directions for further research.

**Reviewer #1:** The immune cell infiltration analysis is a valuable addition to the study, but the interpretation of these results requires more depth. The manuscript should explore the implications of the observed differences in immune cell populations between AP and EBV-infected individuals and controls, and how these findings align with existing knowledge of immune responses in these conditions. Additionally, the correlation analysis between DEGs and immune cell populations could be expanded to include a discussion of potential mechanisms underlying these associations.

**Response:**

Thank you very much for your comments and professional advice. The discussion on the immune cell infiltration analysis has been supplemented in the fifth paragraph of the Discussion section. The results from immune cell infiltration analysis indicated that multiple immune cell populations were more abundant in AP samples than in controls, such as neutrophil and dendritic cells (DCs). Neutrophils are the first-line cells that migrate into the pancreatic inflammation site^[49]^, and are central to the development of pancreatitis, mediating local tissue damage in the pancreas as well as distant organ damage and subsequent death^[50],^^[51]^. Therapeutic interventions targeting neutrophils reduce tissue damage significantly and protect against the development of pancreatitis^[52]^. DCs are known to be essential for pancreatitis recovery^[53]^. Specifically, numbers of MHCII and CD11c dual-positive DCs increased 100-fold in pancreas of AP mice. Intrapancreatic DCs expressed higher levels of MHCII and CD86 and increased production of IL-6, membrane cofactor protein-1, and tumor necrosis factor-α. Depletion of DCs from mice with pancreatitis resulted in neutrophil infiltration and increased levels of systemic markers of inflammation, and died from acinar cell death within four days. A recent study distinguished six subsets of DCs in the pancreas and provided insights into their temporal behavior during pancreatitis^[54]^. DCs are a heterogeneous population and functional characterization of specific DCs subsets may advance our understanding of cell states and responses in pancreatitis and recovery. Further work on this important issue is needed. In addition, we found a lower abundance of activated B cells in AP samples than in controls. The contribution of B cells to tissue injury and regeneration has been investigated in several experimental models^[55],^^[56],^^[57]^. Studies have shown that B cells suppress pancreatic regeneration, and their trafficking and/or function are modulated by hypoxia and HIF1α in the setting of pancreatitis^[58]^. Interestingly, HIF1A has also been identified in the TF-miRNA-mRNA network, which may provide new insights into the precise mechanism. EBV is commonly transmitted through infected saliva and infects resting B lymphocytes and epithelial cells^[59]^. EBV-infected naïve B lymphocytes undergo a germinal center-like activation and differentiation program^[60]^. Furthermore, antigen-presenting cells present antigens to T cells. The generation of EBV-specific CD8+ and CD4+ T cells is markedly elevated in individuals infected with EBV. Infected B cells are then attacked by cytotoxic T lymphocytes (CTLs), which control the number of infected B cells and play antiviral roles^[61]^. Prior research indicated a positive correlation between CD8+ lymphocytosis and disease severity^[62]^. Meanwhile, the eight patients in the dataset GSE45918 are all in serious condition (see original article Supporting Information Table S1). Taken together, this is consistent with our immune infiltration analysis results, where we observed an increase in the abundance of activated CD8 and CD4 T cells and a decrease in activated B cells in the acute EBV- infected samples.

Additionally, we delved into the correlation between the 10 DEGs within miRNA-mRNA network and significantly different immune cell populations in both datasets. E2F2^[63]^, UBE2C^[64]^, LMNB1^[65]^ and CEP55^[66],^^[67]^ are all up-regulated genes and are closely related to cell cycle regulation; cell cycle perturbation has been studied in the context of EBV infection^[68]^ and AP^[69]^, which may partly explain why they are significantly correlated with immune cells with differential abundance in the two groups.

**Reviewer #1:** Regarding the drug prediction analysis, while the identification of histone deacetylase (HDAC) inhibitors as potential therapeutic agents is intriguing, the manuscript would benefit from a more nuanced discussion of the limitations of this approach. The authors should consider the potential challenges in translating these findings into clinical practice, including issues related to drug specificity, off-target effects, and the need for validation in experimental models.

**Response:**

Thank you very much for your comments and professional advice. The discussion on drug prediction analysis has been supplemented in the sixth paragraph of the Discussion section. The basic concept of CMap is to use a reference database containing drug-specific gene expression profiles and compare it with a disease-specific gene signature, whose overall goal is to predict potentially therapeutic drug candidates. However, CMap also has certain constraints, such as limited drug perturbation data, a limited drug coverage, dosage-dependent conditions and the uncertainty of applying cell lines or animal model expression patterns to human systems^[70]^. For more comprehensive understanding the drug MoA, specificity, and off-target effects of HDAC inhibitors, some methodologies incorporating other omics than transcriptomics would be beneficial, including, for instance, methylation array, metabolomics and proteomics, as well as dynamic or longitudinal data, would widen the limited view captured by the single time point of transcriptomic responses, which will facilitate a shift in drug discovery towards a personalized and precision medicine treatment approach.

**Reviewer #2**

**Reviewer #2:** As a reviewer, I have carefully examined the manuscript titled "Exploration of a miRNA-mRNA network shared between acute pancreatitis and Epstein-Barr virus infection by integrated bioinformatics analysis". Here is my detailed review:

This manuscript is recommended for publication after minor revisions. The authors should address the following points:

Discuss plans for experimental validation of key findings in future studies.

**Response:**

Thank you for your comments. In our pursuit of elucidating the intricate relationship between AP and EBV infection, we are undertaking comprehensive etiological screening and pathogen testing of AP patients to determine whether they are related to EBV infection. Concurrently, we intend to conduct a prospective follow-up study to monitor the EBV infection and the occurrence of AP. We will establish a cohort of patients with pancreatitis caused by EBV infection, record their general information and auxiliary examination results, and collect serum, blood cells, urine, and pancreatic tissues (if possible) to establish a biobank. Moreover, we plan to develop an animal model of EBV-related pancreatitis, which will serve as a critical platform for probing the underlying pathological mechanisms and delineating the landscape of immune cells.

**Reviewer #2:** Provide a more detailed rationale for the selection of specific datasets used in the analysis.

**Response:**

Thank you very much for your comments and professional advice. Detailed rationale for the selection of specific datasets used in the analysis has been supplemented in the Data collection section. The criteria for dataset selection were as follows: the dataset must contain samples from both the disease group and the control group; the tissue used for sequencing in different datasets should be as consistent as possible; if there are several homogeneous datasets, the dataset with the largest sample size is preferred.

**Reviewer #2:** Address the potential limitations of using peripheral blood data for understanding pancreatic pathology.

**Response:**

Thank you very much for your comments. Pancreatic tissue is the best candidate for understanding pancreatic pathology during AP. During the datasets screening process, we also found another dataset GSE109227 related with AP in the GEO website. In the dataset GSE109227^[71]^, RNA from cerulein induced AP mice pancreas was extracted and processed for microarray analysis and the authors used these tissues for screening new potential biomarkers of AP. Finally, they identified RCAN1 as a potential diagnostic marker of AP. Since there is no expression profile data of pancreas with EBV infection, and in order to make the tissues used for sequencing in different datasets as consistent as possible, the dataset GSE109227 was omitted. Consistent with our enrichment analysis results, studies revealed that AP mice were enriched in biological processes related to immune system pathways and inflammatory responses^[72],^^[73]^. The results of immune infiltration analysis were also consistent with previous studies^[74],^^[75]^. Studies combining peripheral blood and glomerular data reveal novel diagnostic markers for IgA nephropathy^[76]^, suggesting that peripheral blood cells could be an alternative candidate to study disease pathology. The peripheral blood cells are usually involved in the immune response to EBV and are also involved in the development and progression of pancreatitis. In the present study, we aim to investigate molecules shared in the both disease states. Ultimately, we revealed a miRNA-mRNA network shared between AP and EBV infection, thereby enriching a deeper comprehension of the intricate molecular mechanisms and providing a reference for further research.

**Reviewer #2:** Correct minor typographical errors and ensure consistent formatting.

**Response:**

Thank you very much for your professional advice. The typographical errors and consistent formatting of gene symbols throughout the manuscript have been improved with our careful revision.

**Reviewer #3**

**Reviewer #3:** I feel that this manuscript is very poor. I believe that it lacks proper research value and should not be accepted. The reasons are as follows:

1.In the introduction section, the author proposed that the occurrence of AP is related to EBV infection, and 90% of IM is caused by EBV virus. However, the relationship between AP and IM was not clearly explained. Moreover, the author did not clarify the research significance of TF-miRNA-mRNA.

**Response:**

Thank you very much for your comments. Clinical evidence underscores a robust correlation between EBV infection and AP development. In addition, approximately 90% of IM cases attributed to EBV infection, which is characterized by swollen lymph nodes, fever, sore throat, and often extreme fatigue. Cases of IM complicated with AP have also been reported, some of which have been clearly related to EBV infection. The pancreas is likely one of the target organs affected by the immune response during infection, which leads to the occurrence of pancreatitis. However, these studies are just case reports and do not explore the specific mechanisms of the underlying associations.

Moreover, TF is a master transcriptional regulator that affects the level of miRNA or mRNA. The research significance and biological relevance of the TF-miRNA-mRNA has been supplemented in the fourth paragraph of the Discussion section.

2.There is a question, is the data source of GSE109220 from IM pathological specimens caused by EBV?

**Response:**

Thank you very much for your question. In the dataset GSE109220, Kaul et al. reported the expression of host miRNAs in acute IM caused by primary EBV infection^[77]^. So the answer is yes.

3.Dataset GSE42455 from AP rat models, which is inconsistent with the other three sets of data from humans, resulting in all subsequent analysis results having no scientific basis.

**Response:**

Thank you very much for your question. Animal models, including the rat, have been used for decades to study physiological and pathophysiological mechanisms^[78],^^[79],^^[80],^^[81]^. Many studies combining animal and human data have made outstanding contributions to the discovery of disease-related biomarkers^[82],^^[83],^^[84],^^[85]^. Therefore, our study has a certain scientific basis. Our study presents for the first time a miRNA-mRNA network shared by AP and EBV infections, and enumerates in detail the close association of these molecules with the two pathological states in the Discussion section, which serves as a reference for subsequent studies.

1. Love MI, Huber W, Anders S. Moderated estimation of fold change and dispersion for RNA-seq data with DESeq2. Genome Biol. 2014;15(12):550.
2. Ritchie ME, Phipson B, Wu D, et al. limma powers differential expression analyses for RNA-sequencing and microarray studies. Nucleic Acids Res. 2015;43(7):e47.
3. Luo Y, Zhang Z, Xi S, Li T. Bioinformatics analyses and experimental validation of ferroptosis-related genes in bronchopulmonary dysplasia pathogenesis. PLoS One. 2024;19(6):e0291583.
4. Tao Y, Wei X, Yue Y, et al. Extracellular vesicle-derived AEBP1 mRNA as a novel candidate biomarker for diabetic kidney disease. J Transl Med. 2021;19(1):326.
5. Nesvaderani M, Dhillon BK, Chew T, et al. Gene Expression Profiling: Identification of Novel Pathways and Potential Biomarkers in Severe Acute Pancreatitis. J Am Coll Surg. 2022;234(5):803-815.
6. Zhong X, Shang J, Zhang R, et al. Explore the shared molecular mechanism between dermatomyositis and nasopharyngeal cancer by bioinformatic analysis. PLoS One. 2024;19(5):e0296034.
7. Xu J, Shen X, Wei X, et al. Identification of blood-based key biomarker and immune infiltration in Immunoglobulin A nephropathy by comprehensive bioinformatics analysis and a cohort validation. J Transl Med. 2022;20(1):145.
8. Ru Y, Kechris KJ, Tabakoff B, et al. The multiMiR R package and database: integration of microRNA-target interactions along with their disease and drug associations. Nucleic Acids Res. 2014;42(17):e133.
9. Shaik NA, Nasser K, Mohammed A, et al. Identification of miRNA-mRNA-TFs regulatory network and crucial pathways involved in asthma through advanced systems biology approaches. PLoS One. 2022;17(10):e0271262.
10. Wu T, Hu E, Xu S, et al. clusterProfiler 4.0: A universal enrichment tool for interpreting omics data. Innovation (Camb). 2021;2(3):100141.
11. Kern F, Fehlmann T, Solomon J, et al. miEAA 2.0: integrating multi-species microRNA enrichment analysis and workflow management systems. Nucleic Acids Res. 2020;48(W1):W521-W528.
12. Dunmire SK, Odumade OA, Porter JL, et al. Primary EBV infection induces an expression profile distinct from other viruses but similar to hemophagocytic syndromes. PLoS One. 2014;9(1):e85422.
13. Zheng P, Li XY, Yang XY, et al. Comparative transcriptomic analysis reveals the molecular changes of acute pancreatitis in experimental models. World J Gastroenterol. 2024;30(14):2038-2058.
14. Jangra S, Yuen KS, Botelho MG, Jin DY. Epstein-Barr Virus and Innate Immunity: Friends or Foes?. Microorganisms. 2019;7(6):183.
15. Zhang T, Gan Y, Zhu S. Association between autophagy and acute pancreatitis. Front Genet. 2023;14:998035.
16. Shao Z, Borde C, Quignon F, Escargueil A, Maréchal V. Epstein-Barr Virus BALF0 and BALF1 Modulate Autophagy. Viruses. 2019;11(12).
17. Ylä-Anttila P, Gupta S, Masucci MG. The Epstein-Barr virus deubiquitinase BPLF1 targets SQSTM1/p62 to inhibit selective autophagy. Autophagy. 2021;17(11):3461-74.
18. Epstein-Barr virus-encoded microRNAs as regulators in host immune responses. Int J Biol Sci. 2018;14(5):565-76.
19. Fitzsimmons L, Kelly GL. EBV and Apoptosis: The Viral Master Regulator of Cell Fate? Viruses. 2017;9(11).
20. Bhatia M. Apoptosis versus necrosis in acute pancreatitis. American journal of physiology Gastrointestinal and liver physiology. 2004;286(2):G189-G96.
21. Li JH, Liu S, Zhou H, Qu LH, Yang JH. starBase v2.0: decoding miRNA-ceRNA, miRNA-ncRNA and protein-RNA interaction networks from large-scale CLIP-Seq data. Nucleic Acids Res. 2014;42(Database issue):D92-D97.
22. Wang T, Xu S, Zhang L, et al. Identification of immune-related lncRNA in sepsis by construction of ceRNA network and integrating bioinformatic analysis. BMC Genomics. 2023;24(1):484.
23. Wang A, Chen P. Comprehensive analysis of circRNA-miRNA-mRNA network related to angiogenesis in recurrent implantation failure. BMC Med Genomics. 2024;17(1):193.
24. Liu J, Sun Y, Zhu B, et al. Identification of a potentially novel LncRNA-miRNA-mRNA competing endogenous RNA network in pulmonary arterial hypertension via integrated bioinformatic analysis. Life Sci. 2021;277:119455.
25. Xu J, Shen X, Wei X, et al. Identification of blood-based key biomarker and immune infiltration in Immunoglobulin A nephropathy by comprehensive bioinformatics analysis and a cohort validation. J Transl Med. 2022;20(1):145. doi:10.1186/s12967-022-03330-w
26. Saini J, Thapa U, Bandyopadhyay B, Vrati S, Banerjee A. Knockdown of NEAT1 restricts dengue virus replication by augmenting interferon alpha-inducible protein 27 via the RIG-I pathway. J Gen Virol. 2023;104(1).
27. Yang Y, Li M, Ma Y, Ye W, Si Y, Zheng X, et al. LncRNA NEAT1 Potentiates SREBP2 Activity to Promote Inflammatory Macrophage Activation and Limit Hantaan Virus Propagation. Front Microbiol. 2022;13:849020.
28. Shao A, Hu W, Li C, Yang Y, Zhu J. Downregulation of lncRNA NEAT1 Relieves Caerulein-Induced Cell Apoptosis and Inflammatory Injury in AR42J Cells Through Sponging miR-365a-3p in Acute Pancreatitis. Biochem Genet. 2022;60(6):2286-98.
29. Xia TF, Chen J, Wu K, Zhang J, Yan Q. Long noncoding RNA NEAT1 promotes the growth of gastric cancer cells by regulating miR-497-5p/PIK3R1 axis. Eur Rev Med Pharmacol Sci. 2019;23(16):6914-26.
30. Gao C, Sun J, Zhang Z, Xu Z. NEAT1 Boosts the Development of Thoracic Aortic Aneurysm Through Targeting miR-324-5p/RAN. Arch Med Res. 2022;53(1):93-9.
31. Luan L, Hu Q, Wang Y, Lu L, Ling J. Knockdown of lncRNA NEAT1 expression inhibits cell migration, invasion and EMT by regulating the miR-24-3p/LRG1 axis in retinoblastoma cells. Exp Ther Med. 2021;21(4):367.
32. Yan Q, Hu Q, Li G, Qi Q, Song Z, Shu J, et al. NEAT1 Regulates Calcium Oxalate Crystal-Induced Renal Tubular Oxidative Injury via miR-130/IRF1. Antioxid Redox Signal. 2023;38(10-12):731-46.
33. The Epstein-Barr virus protein BRLF1 activates S phase entry through E2F1 induction. J Virol. 1999;73(8):6540-50.
34. Mauser A, Holley-Guthrie E, Zanation A, Yarborough W, Kaufmann W, Klingelhutz A, et al. The Epstein-Barr virus immediate-early protein BZLF1 induces expression of E2F-1 and other proteins involved in cell cycle progression in primary keratinocytes and gastric carcinoma cells. J Virol. 2002;76(24):12543-52.
35. Lin S, Qiu L, Liang K, Zhang H, Xian M, Chen Z, et al. KAT2A/E2F1 Promotes Cell Proliferation and Migration via Upregulating the Expression of UBE2C in Pan-Cancer. Genes (Basel). 2022;13(10).
36. Zhao L, Zheng Y, Zhang L, Su L. E2F1-Induced FTH1P3 Promoted Cell Viability and Glycolysis Through miR-377-3p/LDHA Axis in Laryngeal Squamous Cell Carcinoma. Cancer Biother Radiopharm. 2022;37(4):276-86.
37. Su X-J, Chen Y, Zhang Q-C, Peng X-B, Liu Y-P, Wang L, et al. Exosomes Derived From Cerulein-Stimulated Pancreatic Acinar Cells Mediate Peritoneal Macrophage M1 Polarization and Pyroptosis via an miR-24-3p/MARCH3/NLRP3 Axis in Acute Pancreatitis. Pancreas. 2024;53(8):e641-e51.
38. Geng X, Zou Y, Li S, Qi R, Yu H, Li J. MALAT1 Mediates α-Synuclein Expression through miR-23b-3p to Induce Autophagic Impairment and the Inflammatory Response in Microglia to Promote Apoptosis in Dopaminergic Neuronal Cells. Mediators Inflamm. 2023;2023:4477492.
39. Yang X, Yang X, Sun A, Chen S, Wang X, Zhao X. The miR-23b-3p from adipose-derived stem cell exosomes alleviate inflammation in mice experiencing kainic acid-induced epileptic seizures. Neuroreport. 2024;35(10):612-20.
40. Wang Q, Wang H, Jing Q, Yang Y, Xue D, Hao C, et al. Regulation of Pancreatic Fibrosis by Acinar Cell-Derived Exosomal miR-130a-3p via Targeting of Stellate Cell PPAR-γ. J Inflamm Res. 2021;14:461-77.
41. Ding Y, Hou Y, Liu Y, Yu T, Cui Y, Nie H. MiR-130a-3p Alleviates Inflammatory and Fibrotic Phases of Pulmonary Fibrosis Through Proinflammatory Factor TNF-α and Profibrogenic Receptor TGF-βRII. Front Pharmacol. 2022;13:863646.
42. Zhang Z, Zhao T, Xu H, Wu X. Circ_0008365 Suppresses Apoptosis, Inflammation and Extracellular Matrix Degradation of IL-1β-treated Chondrocytes in Osteoarthritis by Regulating miR-324-5p/BMPR2/NF-κB Signaling Axis. Immunol Invest. 2022;51(6):1598-611.
43. Zhang D, Chen X, Zheng D. A Novel MIR503HG/miR-497-5p/CCL19 Axis Regulates High Glucose-Induced Cell Apoptosis, Inflammation, and Fibrosis in Human HK-2 Cells. Appl Biochem Biotechnol. 2022;194(5):2061-76.
44. Su M, Chen F, Han D, Song M, Wang Y. PRMT7-Dependent Transcriptional Activation of Hmgb2 Aggravates Severe Acute Pancreatitis by Promoting Acsl1-Induced Ferroptosis. J Proteome Res. 2024;23(3):1075-87.
45. Huang D-N, Zhong H-J, Cai Y-L, Xie W-R, He X-X. Serum Lactate Dehydrogenase Is a Sensitive Predictor of Systemic Complications of Acute Pancreatitis. Gastroenterol Res Pract. 2022;2022:1131235.
46. Cui J, Xiong J, Zhang Y, Peng T, Huang M, Lin Y, et al. Serum lactate dehydrogenase is predictive of persistent organ failure in acute pancreatitis. J Crit Care. 2017;41:161-5.
47. Li H, Xie J, Guo X, Yang G, Cai B, Liu J, et al. Bifidobacterium spp. and their metabolite lactate protect against acute pancreatitis via inhibition of pancreatic and systemic inflammatory responses. Gut Microbes. 2022;14(1):2127456.
48. Tang G-X, Yang M-S, Xiang K-M, Yang B-C, Liu Z-L, Zhao S-P. MiR-20b-5p modulates inflammation, apoptosis and angiogenesis in severe acute pancreatitis through autophagy by targeting AKT3. Autoimmunity. 2021;54(7):460-70.
49. Shamoon M, Deng Y, Chen YQ, Bhatia M, Sun J. Therapeutic implications of innate immune system in acute pancreatitis. Expert Opin Ther Targets. 2016;20(1):73-87.
50. Fukuda Y, Mori K, Okada H, Tomita H, Suzuki K, Takada C, et al. Decreased neutrophil counts prolong inflammation in acute pancreatitis and cause inflammation spillover to distant organs. Pancreatology. 2023;23(8):911-8.
51. Ishqi HM, Ali M, Dawra R. Recent advances in the role of neutrophils and neutrophil extracellular traps in acute pancreatitis. Clin Exp Med. 2023;23(8):4107-22.
52. Han F, Ding ZF, Shi XL, Zhu QT, Shen QH, Xu XM, et al. Irisin inhibits neutrophil extracellular traps formation and protects against acute pancreatitis in mice. Redox Biol. 2023;64:102787.
53. Bedrosian AS, Nguyen AH, Hackman M, Connolly MK, Malhotra A, Ibrahim J, et al. Dendritic cells promote pancreatic viability in mice with acute pancreatitis. Gastroenterology. 2011;141(5).
54. Aney KJ, Jeong W-J, Vallejo AF, Burdziak C, Chen E, Wang A, et al. Novel Approach for Pancreas Transcriptomics Reveals the Cellular Landscape in Homeostasis and Acute Pancreatitis. Gastroenterology. 2024;166(6):1100-13.
55. Jang HR, Gandolfo MT, Ko GJ, Satpute SR, Racusen L, Rabb H. B cells limit repair after ischemic acute kidney injury. J Am Soc Nephrol. 2010;21(4):654-65.
56. Zouggari Y, Ait-Oufella H, Bonnin P, Simon T, Sage AP, Guérin C, et al. B lymphocytes trigger monocyte mobilization and impair heart function after acute myocardial infarction. Nat Med. 2013;19(10):1273-80.
57. Novobrantseva TI, Majeau GR, Amatucci A, Kogan S, Brenner I, Casola S, et al. Attenuated liver fibrosis in the absence of B cells. J Clin Invest. 2005;115(11):3072-82.
58. Lee KE, Spata M, Maduka R, Vonderheide RH, Simon MC. Hif1α Deletion Limits Tissue Regeneration via Aberrant B Cell Accumulation in Experimental Pancreatitis. Cell Rep. 2018;23(12):3457-64.
59. Huang W, Bai L, Tang H. Epstein-Barr virus infection: the micro and macro worlds. Virol J. 2023;20(1):220.
60. Mrozek-Gorska P, Buschle A, Pich D, Schwarzmayr T, Fechtner R, Scialdone A, et al. Epstein-Barr virus reprograms human B lymphocytes immediately in the prelatent phase of infection. Proc Natl Acad Sci U S A. 2019;116(32):16046-55.
61. Huang W, Bai L, Tang H. Epstein-Barr virus infection: the micro and macro worlds. Virol J. 2023;20(1):220.
62. Balfour HH, Odumade OA, Schmeling DO, Mullan BD, Ed JA, Knight JA, et al. Behavioral, virologic, and immunologic factors associated with acquisition and severity of primary Epstein-Barr virus infection in university students. J Infect Dis. 2013;207(1):80-8.
63. Infante A, Laresgoiti U, Fernández-Rueda J, et al. E2F2 represses cell cycle regulators to maintain quiescence. Cell Cycle. 2008;7(24):3915-3927.
64. Nicolau-Neto P, Palumbo A, De Martino M, et al. UBE2C Is a Transcriptional Target of the Cell Cycle Regulator FOXM1. Genes (Basel). 2018;9(4):188.
65. Gao D, Guo H, Liu Z, Bao L, Li S, Wang Y, et al. LMNB1/CDKN1A Signaling Regulates the Cell Cycle and Promotes Hepatocellular Carcinoma Progression. Curr Cancer Drug Targets. 2024.
66. Xu Z-Y, Ma X-S, Qi S-T, Wang Z-B, Guo L, Schatten H, et al. Cep55 regulates spindle organization and cell cycle progression in meiotic oocyte. Sci Rep. 2015;5:16978.
67. van der Horst A, Simmons J, Khanna KK. Cep55 stabilization is required for normal execution of cytokinesis. Cell Cycle. 2009;8(22):3742-9.
68. Yin H, Qu J, Peng Q, Gan R. Molecular mechanisms of EBV-driven cell cycle progression and oncogenesis. Med Microbiol Immunol. 2019;208(5):573-83.
69. de Dios I, Uruñuela A, Pinto RM, Orfao A, Manso MA. Cell-cycle distribution of pancreatic cells from rats with acute pancreatitis induced by bile-pancreatic obstruction. Cell Tissue Res. 2000;300(2):307-14.
70. Musa A, Ghoraie LS, Zhang S-D, Glazko G, Yli-Harja O, Dehmer M, et al. A review of connectivity map and computational approaches in pharmacogenomics. Brief Bioinform. 2018;19(3):506-23.
71. Norberg KJ, Nania S, Li X, Gao H, Szatmary P, Segersvärd R, et al. RCAN1 is a marker of oxidative stress, induced in acute pancreatitis. Pancreatology. 2018;18(7):734-41.
72. Zheng P, Li X-Y, Yang X-Y, Wang H, Ding L, He C, et al. Comparative transcriptomic analysis reveals the molecular changes of acute pancreatitis in experimental models. World J Gastroenterol. 2024;30(14):2038-58.
73. Aney KJ, Jeong W-J, Vallejo AF, Burdziak C, Chen E, Wang A, et al. Novel Approach for Pancreas Transcriptomics Reveals the Cellular Landscape in Homeostasis and Acute Pancreatitis. Gastroenterology. 2024;166(6):1100-13.
74. Ishqi HM, Ali M, Dawra R. Recent advances in the role of neutrophils and neutrophil extracellular traps in acute pancreatitis. Clin Exp Med. 2023;23(8):4107-22.
75. Huang W, Bai L, Tang H. Epstein-Barr virus infection: the micro and macro worlds. Virol J. 2023;20(1):220.
76. Xu J, Shen X, Wei X, Ding J, Yuan J, Weng Z, et al. Identification of blood-based key biomarker and immune infiltration in Immunoglobulin A nephropathy by comprehensive bioinformatics analysis and a cohort validation. Journal of Translational Medicine. 2022;20(1):145.
77. Kaul V, Weinberg KI, Boyd SD, et al. Dynamics of Viral and Host Immune Cell MicroRNA Expression during Acute Infectious Mononucleosis. Front Microbiol. 2018;8:2666.
78. Bryda EC. The Mighty Mouse: the impact of rodents on advances in biomedical research. Mo Med. 2013;110(3):207-211.
79. Domínguez-Oliva A, Hernández-Ávalos I, Martínez-Burnes J, Olmos-Hernández A, Verduzco-Mendoza A, Mota-Rojas D. The Importance of Animal Models in Biomedical Research: Current Insights and Applications. Animals (Basel). 2023;13(7):1223.
80. Dwinell MR, Lazar J, Geurts AM. The emerging role for rat models in gene discovery. Mamm Genome. 2011;22(7-8):466-475.
81. Szpirer C. Rat models of human diseases and related phenotypes: a systematic inventory of the causative genes. J Biomed Sci. 2020;27(1):84.
82. Wu Z, Chen X, Zhang K, et al. Identification of Hub Genes in the Pathogenesis of Bronchiolitis Obliterans via Bioinformatic Analysis and Experimental Verification. J Inflamm Res. 2023;16:3303-3317.
83. Yao H, Xiao Z, Liu S, et al. Screening of novel disease genes of sepsis-induced myocardial Disfunction by RNA sequencing and bioinformatics analysis. Genomics. Published online August 5, 2024. doi:10.1016/j.ygeno.2024.110911
84. Tao Y, Wei X, Yue Y, et al. Extracellular vesicle-derived AEBP1 mRNA as a novel candidate biomarker for diabetic kidney disease. J Transl Med. 2021;19(1):326.
85. Lu Y, Li K, Hu Y, Wang X. Expression of Immune Related Genes and Possible Regulatory Mechanisms in Alzheimer's Disease. Front Immunol. 2021;12:768966.
